# Enhanced Photoluminescence of Crystalline Alq_3_ Micro-Rods Hybridized with Silver Nanowires

**DOI:** 10.3390/nano13050825

**Published:** 2023-02-23

**Authors:** Misuk Kim, Jiyoun Kim, Seongcheol Ju, Hyeonwoo Kim, Incheol Jung, Jong Hoon Jung, Gil Sun Lee, Young Ki Hong, Dong Hyuk Park, Kyu-Tae Lee

**Affiliations:** 1 Department of Physics, Inha University, Incheon 22212, Republic of Korea; 2 Department of Chemical Engineering, Program in Biomedical Science and Engineering, Inha University, Incheon 22212, Republic of Korea; 3 Department of General Education, Kookmin University, Seoul 02707, Republic of Korea; 4 Department of Physics, Gyeongsang National University, Jinju 52828, Republic of Korea; 5 Research Institute of Natural Science, Gyeongsang National University, Jinju 52828, Republic of Korea

**Keywords:** Alq_3_, crystalline micro-rod, Ag, nanowire, hybridization, surface plasmon resonance

## Abstract

An enhancement of the local electric field at the metal/dielectric interface of hybrid materials due to the localized surface plasmon resonance (LSPR) phenomenon plays a particularly important role in versatile research fields resulting in a distinct modification of the electrical, as well as optical, properties of the hybrid material. In this paper, we succeeded in visually confirming the LSPR phenomenon in the crystalline tris(8-hydroxyquinoline) aluminum (Alq_3_) micro-rod (MR) hybridized with silver (Ag) nanowire (NW) in the form of photoluminescence (PL) characteristics. Crystalline Alq_3_ MRs were prepared by a self-assembly method under the mixed solution of protic and aprotic polar solvents, which could be easily applied to fabricate hybrid Alq_3_/Ag structures. The hybridization between the crystalline Alq_3_ MRs and Ag NWs was confirmed by the component analysis of the selected area electronic diffraction attached to high-resolution transmission electron microscope. Nanoscale and solid state PL experiments on the hybrid Alq_3_/Ag structures using a lab-made laser confocal microscope exhibited a distinct enhancement of the PL intensity (approximately 26-fold), which also supported the LSPR effects between crystalline Alq_3_ MRs and Ag NWs.

## 1. Introduction

Luminescence, the spontaneous emission of light, arises from the transition of electrons from the highest occupied molecular orbital (HOMO) to the lowest unoccupied molecular orbital (LUMO). Various types of luminescence have been identified, including chemiluminescence, where electron excitation is caused by the energy released in a chemical reaction, electroluminescence, which is a result of the passage of an electric current, and photoluminescence (PL), which is generated by the absorption of photons [1,2,3].

Significant efforts have been dedicated to enhancing the PL efficiency of light-emitting organic materials by exploiting surface plasmon polaritons (SPPs), which are hybridized excitations propagating at the interface between metals and dielectrics when collective electron oscillations (i.e., SPs) couple with photons in metallic nanostructures [4,5,6,7,8]. Matching the energy of the SPPs in the metallic nanostructures with that of the emitted photons of the organic luminescent materials allows resonance to occur, thus leading to a significantly enhanced PL efficiency at the resonant wavelengths [9,10]. The resonant wavelength can be easily tuned by altering the materials, the geometric parameters of the metallic nanostructures, and the surrounding media, etc. [11,12,13,14]. These localized surface plasmon resonance (LSPR) effects have been reported in various research fields, such as color change sensors for lights, DNA sensors, and color barcode nanowire (NW) [9,10,11,13,15,16,17,18].

The optical and electrical properties of tris(8-hydroxyquinoline) aluminum (Alq_3_), which is one of the most widely used organic small molecules in diverse opto-electronic devices [19,20,21], were increased by modifying the chemical structures or changing the crystal structures [22,23,24] Furthermore, it has also been reported that the LSPR phenomena of Alq_3_ and metal nanostructures influence its optical properties [25]. After the report on a crystalline form of the organic small molecules was published, such as rubrene, exhibiting highly stable and increasing PL characteristics [26,27,28,29], a single crystal based on a benzene derivative showed efficient and planar optical waveguiding capabilities, i.e., the lateral propagation of normally incident photon energy, which is very different from the axial waveguiding commonly observed in optical fibers [30]. These reports would imply that the crystalline organic small molecular structure can be an excellent and potential candidate for dielectric counterparts in hybridization, which is essential for the LSPR procedure [18,31,32,33]. 

This paper reports a large enhancement of the LSPR phenomenon in crystalline Alq_3_ micro-rod (MR) hybridized with silver (Ag) NWs, which are denoted as “hybrid Alq_3_/Ag NWs-MR” hereafter. Crystalline Alq_3_ MRs were fabricated by a self-assembly method with the aid of a surfactant, as well as an amphiphilic solvent. For the hybrid Alq_3_/Ag NWs-MRs, surface-functionalized Ag NWs were attached on the surface of the Alq_3_ MR. The formation and hybridization between the crystalline Alq_3_ MRs and Ag NWs were investigated using elementary analyses and high-resolution electron microscopy experiments. The nanoscale and solid state optical properties of the pristine Alq_3_ MRs (i.e., without Ag NWs) and hybrid Alq_3_/Ag NW-MRs were also compared to support the LSPR effects in the developed hybrid systems.

## 2. Materials and Methods

### 2.1. Synthesis of Hybrid Alq_3_/Ag NWs-MRs

Alq_3_ (C_27_H_18_AlN_3_O_3_, purity 99.995%) and cetyltrimethylammonium bromide (CTAB; C_19_H_42_BrN, purity 99.0%) were purchased from Sigma Aldrich (St. Louis, MO, USA) and used without further purification. For the crystalline Alq_3_ MRs, 8 mg of Alq_3_ powder was dissolved in 1 mL of tetrahydrofuran (THF). Next, an aqueous CTAB solution was prepared with a concentration of 1 mg·mL^−1^ in which CTAB acted as a surfactant. Subsequently, an Alq_3_ solution was injected dropwise into an aqueous CTAB solution, resulting in a homogeneously dispersed crystalline Alq_3_ MRs solution due to the amphiphilic nature of the THF, as well as the CTAB surfactants. It should also be noted that only Alq_3_ nanoparticles (NPs) were obtained without a surfactant [10,11,24,34].

For the hybrid Alq_3_/Ag NWs-MRs, 0.5 wt% of the Ag NWs-dispersed aqueous solution was added into 0.5 mL of the Alq_3_ MRs dispersed solution. Ag NWs were fabricated with polyol methods, in which the surface of the each Ag NW was encapsulated by a thin layer of polyvinylpyrrolidone [35,36,37]. Thus, surface-functionalized Ag NWs were compatibly attached onto the surfaces of the crystalline Alq_3_ MRs. The length and diameter of the Ag NW were a few tens of micrometers and approximately 40 nm, respectively.

### 2.2. Formation of Crystalline Hybrid MRs

The surface morphology of the crystalline Alq_3_ MRs was analyzed using field emission scanning electron microscope (FE-SEM; Hitachi, Tokyo, Japan, SU-8010) at an acceleration voltage of 15 kV. The crystalline structures of the pristine and hybrid MRs were analyzed using a high-resolution transmission electron microscope (HR-TEM; Tecnai G2, Fei) with an acceleration voltage of 200 kV and selected area electron diffraction (SAED). X-ray diffraction (XRD; X’Pert Powder Diffractometer, PANalytical) patterns were acquired at a voltage of 40 kV and current of 40 mA using Cu-Kα radiation (*λ* = 1.540 Å). The scan rate was 0.02 degree·s^−1^, and the 2*θ* range was 2−80°. Luminescent color charge-coupled device (CCD) images were acquired using an AVT Marlin F-033C (*λ*_ex_ = 435 nm) instrument.

### 2.3. Photoluminescences of Crystalline Hybrid MRs

The PL spectra were acquired using a lab-made laser confocal microscope (LCM) instrument. The 405 nm line of an unpolarized diode laser was used to excite the samples. The crystalline Alq_3_ MRs were placed on a glass substrate mounted on the XY stage of the LCM. An oil immersion objective lens (N.A. of 1.4) was used to focus the unpolarized laser light on the samples with a spot size of approximately 200 nm. The scattered light was collected using the same objective lens, and the excitation laser light was filtered out through a long-pass edge filter (Semrock, Rochester, NY, USA). The red-shifted PL signal was focused onto a multimode fiber (core size = 50 μm), which acted as a pinhole for confocal detection. The other end of the multimode fiber was connected to a photomultiplier tube for the acquisition of the PL image or the input slit of a 0.3 m long monochromator equipped with a cooled CCD for acquisition of the PL spectra. To compare the brightness (i.e., luminescence intensity) of the CCD images of the pristine and hybrid MRs, the irradiation time was fixed at 0.1 s. For a quantitative comparison, the incident laser power and acquisition time for each LCM PL spectrum were fixed at 5 μW and 1 s, respectively, in all the LCM PL measurements [38,39]. For statistical justification, more than 20 spectra for the pristine and hybrid sample were averaged by using data plotting software (Gnuplot Ver 5.4).

## 3. Results and Discussion

**Figure 1:**
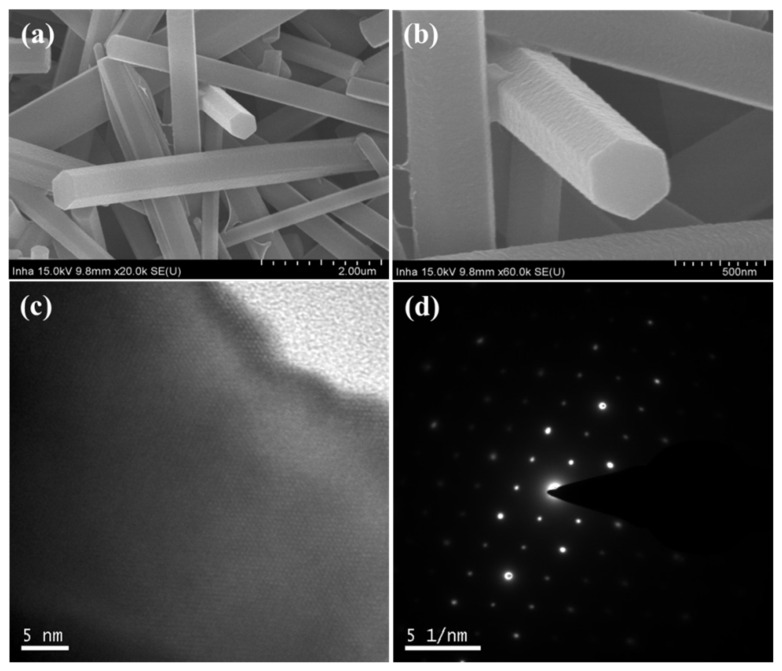
(**a**,**b**) show SEM images of the pristine Alq_3_ MRs with different magnifications; (**c**) HR-TEM image of the single crystalline Alq_3_ MR. (**d**) Corresponding SAED pattern of (**c**).

The shapes and surface morphologies of the pristine Alq_3_ MRs and hybrid Alq_3_/Ag NWs-MRs were confirmed through SEM experiments. The crystallinity and structural characteristics were verified using HR-TEM and SAED experiments. The pristine Alq_3_ MRs exhibited a uniform and continuous one-dimensional array, with a mean length of approximately 10 μm, as shown in the side view SEM image in Figure 1a. We observed that the pristine Alq_3_ MRs exhibited a hexagonal cross-section, with a diameter of 0.5−1 μm, as shown in the magnified SEM image in Figure 1b. From the crystalline lattice image of the HR-TEM shown in Figure 1c, we can conclude that the Alq_3_ MRs were well grown in a crystalline form. The SAED pattern in Figure 1d also demonstrates the intrinsic hexagonal crystallinity of Alq_3_.

**Figure 2:**
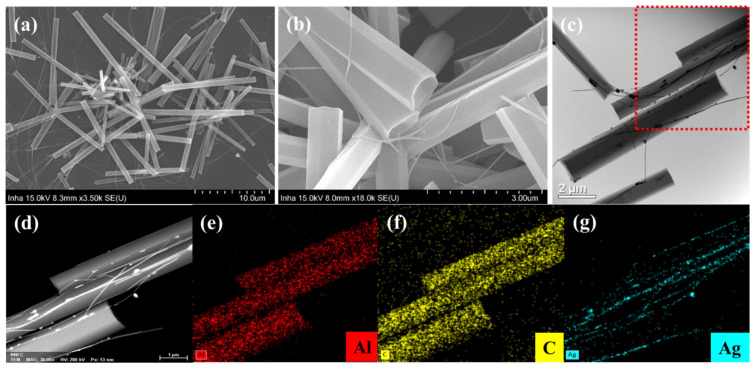
(**a**,**b**) show SEM images of the hybrid Alq_3_/Ag NWs-MRs. (**c**) HR-TEM image of a single hybrid Alq_3_/Ag NWs-MR. (**d**–**g**) EDX mapping images in the red dashed box of (**c**), exhibiting Al (**e**), C (**f**), and Ag (**g**), respectively.

To obtain more convincing evidence of the hybridization of the Alq_3_ MRs and Ag NWs, the morphology of the hybrid Alq_3_/Ag NWs-MRs was carefully investigated using SEM, as shown in Figure 2a,b and the HR-TEM in Figure 2c. The surface-functionalized Ag NWs were intertwined with the crystalline Alq_3_ MRs, which can be well observed in Figure 2b,d. HR-TEM acquires images through the transmitted electrons, which facilitated the observation of the coexistence of two different materials with distinct electron transmissions characteristics. As shown in Figure 2c, the Ag part in the hybrid MR is relatively dark due to the low electron transmission, whereas the Alq_3_ part with good transmission is brighter than the Ag NWs are. Figure 2d–g present the energy-dispersive X-ray spectroscopic (EDX) mapping experiments conducted in the red, dashed box in Figure 2c, which shows the primary element distribution. The red color in Figure 2e, the yellow in Figure 2f, and the blue in Figure 2g denote aluminum (Al), carbon (C), and Ag, respectively. Three strands of the Alq_3_ MRs can be identified in Figure 2e,f. In addition, Al and C were homogeneously distributed in each strand of the MR. The blue color distribution of Ag matches well with the shape of the Ag NW attached on the crystalline Alq_3_ MRs, as shown in Figure 2g.

**Figure 3:**
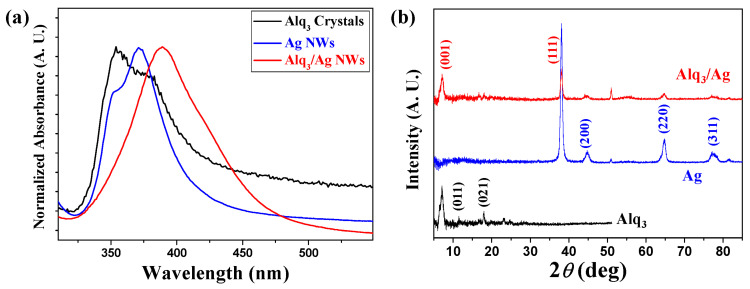
Comparison of the (**a**) UV absorption spectra and (**b**) XRD patterns of the pristine Alq_3_ MRs (black), Ag NWs (blue), and hybrid Alq_3_/Ag NWs-MRs (red), respectively.

Figure 3a shows the normalized ultraviolet (UV)-visible absorption spectra of the Ag NWs, pristine Alq_3_ MRs, and hybrid Alq_3_/Ag NWs-MRs. In the absorption spectra of the Ag NWs and pristine Alq_3_ MRs, absorption peaks were observed at 355 and 371 nm, respectively. 

It should be noted that there exists a relatively large overlap in the absorption spectrum of the pristine Alq_3_ MRs with that of the Ag NWs, which are beneficial for energy transfer between them. The absorption at 355 nm due to the π-π* transition plays an important role in the bright green emission of Alq_3_, which corresponds to the plasmon band energy of Ag (*E*_g_ = 3.34 eV). Owing to the good matching of the energy levels, the interaction between Alq_3_ and Ag contributes to the LSPR phenomenon. The spectrum of the hybrid Alq_3_/Ag NWs-MRs showed an absorption peak at 389 nm. Compared with the intrinsic absorption of Ag NWs and Alq_3_ MRs, the spectrum of the hybrid Alq_3_/Ag NWs-MRs exhibited a relatively red shift, and a broader full width at half maximum because of the strong interaction between Alq_3_ and Ag during LSPR coupling [5,9,40,41]. 

The XRD analysis shown in Figure 3b was performed as another method to determine the crystalline properties of the samples. Since the distances between the atoms and lattice structures are different depending on the materials, Miller indices were evaluated using Bragg’s diffraction law and were compared with the measured 2*θ* values obtained using the XRD equipment. The black line in Figure 3b shows the XRD data of the pristine Alq_3_ MRs. Alq_3_ has *d* spacing values of 13.79, 7.68, and 4.97 Å, which were associated with the lattice planes of the (001), (011), and (021) directions, respectively. According to these XRD peaks, we can find that crystalline Alq_3_ MRs grew with a typical pattern for α-Alq_3_ [20]. In the case of Ag NWs, the XRD result shows that the *d* spacing values were 2.36, 2.05, 1.45, and 1.22 Å and their lattice planes were (111), (200), (220), and (311), respectively, as seen from the blue line of Figure 3b [37,42]. In the hybrid Alq_3_/Ag NWs-MRs, we can find the XRD patterns of each material such as 2*θ* values of 6.4, 11.5, 17.8, 38, 44, and 64°, as shown in the red line of Figure 3b. These values of the hybrid Alq_3_/Ag NWs-MRs coincide with individual XRD results of the Alq_3_ and Ag, indicating the successful completion of hybridization in the crystalline form. 

**Figure 4:**
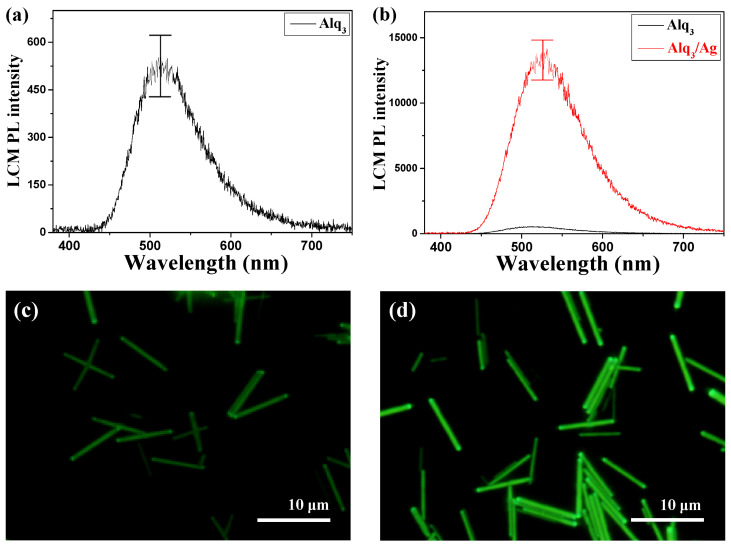
(**a**) PL spectrum of the pristine Alq_3_ MRs. (**b**) Comparison of PL spectra of the pristine Alq_3_ MRs and hybrid Alq_3_/Ag NWs-MRs. (**c**,**d**) show the corresponding color CCD images of the pristine Alq_3_ MRs (**a**) and hybrid Alq_3_/Ag NWs-MRs (**b**), respectively.

Figure 4a presents the PL spectrum of a single pristine Alq_3_ MR. The maximum PL peak was observed at 511 nm, which is in good agreement with the relatively weak green light shown in Figure 4c. In Figure 4b, the PL intensities are compared with the hybrid Alq_3_/Ag NWs-MR and pristine Alq_3_ MR. The maximum PL peak of the hybrid Alq_3_/Ag NWs-MRs was at 524 nm, which was slightly more red shifted compared to that of the pristine Alq_3_ MR. However, the PL intensity of the hybrid Alq_3_/Ag NWs-MR dramatically increased by approximately 26-fold. From the color CCD attached to LCM equipment, the luminescence images of the pristine Alq_3_ MRs and hybrid Alq_3_/Ag NWs-MR were directly obtained, as shown in Figure 4c,d, respectively. The luminescence image of the pristine Alq_3_ MRs exhibited a weak green emission, whereas that of the hybrid Alq_3_/Ag NWs-MRs was significantly brighter than the pristine ones were. When the plasmon activation energy of Ag NWs and the optical absorption energy of Alq_3_ are well matched and harmonized, a plasmon resonance interaction occurs, and excited excitons are formed by the collective excitation of electrons [9,10,11,40,41]. Consequently, the emission efficiency of the hybrid Alq_3_/Ag NWs-MRs was significantly increased, which could confirm the LSPR phenomenon through the improvement of the LCM PL intensities and brightness of the color CCD images. For quantitative comparison, the structural characteristics and optical features of the various organic/metal hybrid structures exhibiting a distinct PL intensity enhancement are listed in Table 1. Undoped poly (2-methoxy-5-(2′-ethylhexyloxy)-*p*-phenylene vinylene) (MEH-PPV) hybridized with gold (Au) NPs showed an approximate 30-fold enhancement of the PL intensity without a PL peak shift because the LSPR only results in the resonant energy transfer between MEH-PPV and Au [11]. However, PL peaks of the hybrid structures consisting of the doped polymeric nanostructures, e.g., electrochemically synthesized nanotubes (NTs) or NWs, were significantly increased and red shifted [4,5]. These results are attributed that the LSPR influences on both energy and charge transfer effect [9,10]. For the undoped organic small molecules, the enhancement of the PL intensity are only caused by the energy transfer effect. Therefore, it is very important to precisely control the crystalline nature of light-emitting organic small molecular materials, as well as metallic nanomaterials.

## 4. Conclusions

Ag and Alq_3_, which are well matched with the plasmon excitation energy of metals and the band gap of organic semiconductors, were hybridized to improve the luminescent properties using the LSPR phenomenon. HR-TEM and SAED observations confirmed that Alq_3_ grew in a crystalline form. The hybrid formation of the Ag NWs and crystalline Alq_3_ MRs was confirmed through EDX and XRD analyses. The strength of the PL and color CCD images of the hybrid Alq_3_/Ag NWs-MRs measured in the LCM PL equipment increased by approximately 26-fold compared to that of the pristine MRs, and they were significantly brighter. This indicated that strong LSPR coupling occurred. Future work would focus on the development of nanocomposite materials, where hybrid Alq_3_/Ag NWs-MRs are embedded in dielectric matrices for the further enhancement of the photoluminescence by the LSPR and local field effects [43,44].

## Figures and Tables

**Table 1:** Comparison of the structural characteristics and typical PL features in the various hybrid organic/metal structures.

Organic Materials	Type	Metal	Type	PL Peak Shift	PL Enhancement (Fold)	Refs.
Alq_3_	MR	Ag	NWs	X	~26	This Work
Alq_3_	NW	Ag	NPs	X	4–5	[19]
Rubrene	1-dimensional MR	Au	NPs	X	~2	[18]
MEH-PPV	NP	Au	NPs	X	~30	[11]
Polythiophene	NT	Co/Cu/Ni	NT	O	40–70	[4]
Poly (3-methylthiophene)	NT	Ni	NT	O	~350	[5]

## Data Availability

The data presented in this study are available upon request from the corresponding authors.
